# Letter from the Editor-in-Chief

**DOI:** 10.19102/icrm.2017.080705

**Published:** 2017-07-15

**Authors:** Moussa Mansour


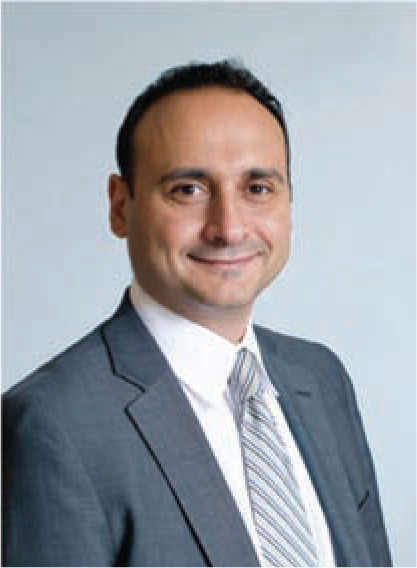


Dear Readers,

This issue of *The Journal of Innovations in Cardiac Rhythm Management* contains several interesting articles. I would like to highlight the one by Robinson and colleagues titled “Confluent Extended Posterior Left Atrial Wall Ablation: Thinking Inside the Box.” In it, the authors describe their experience with the hybrid endocardial and epicardial ablation procedure for persistent and long-standing persistent atrial fibrillation (AF). Fifty-seven patients were enrolled and followed up with for an average of 488 days in this study, with a single procedure success rate of 59%.

This article is important because it highlights the issue of patients requiring adjunctive ablations in combination with pulmonary (PV) isolation, in those with persistent and longstanding persistent AF. While PV isolation is an effective treatment for use in paroxysmal and some early persistent AF patients, it is generally accepted that adjunctive ablation strategies are needed for the successful management of more advanced forms of AF. The posterior left atrial (LA) wall has been shown in many clinical and pre-clinical studies to harbor triggers/drivers of AF. As a result, strategies targeting this area of the left atrium are in theory expected to improve the success rate of the procedure. The epicardial convergent procedure provides an effective way to ablate the posterior LA wall.

The hybrid procedure however, in its current form, is associated with some limitations including significant morbidity with the surgical epicardial approach, when compared with the less-invasive endocardial approach, and with respect to the long duration of the procedure. Patients often have higher levels of pain after the procedure, and stay longer in the hospital. On the other hand, the posterior LA wall can easily be ablated endocardially in a short amount of time. Following wide-area antral isolation, which is a standard practice in most electrophysiology labs, the ablation of the posterior LA wall can be achieved with only a few additional minutes of ablation. Esophageal heating and injury are significant problems associated with the endocardial approach; however, these complications can be potentially eliminated with novel tools, allowing for the deviation of the esophagus away from the field of ablation. As a result, the epicardial surgical technique appears to represent a costly intervention to use if performed for the sole reason of ablating the posterior LA wall, an endpoint that can also be achieved fairly easily with a catheter endocardially. However, the epicardial surgical approach can be made more valuable if it includes the closure and silencing of the LA appendage. More and more studies are implicating this structure in the maintenance of persistent AF. Clipping the LA appendage epicardially may improve the success of the AF ablation procedure, and may allow for the cessation of oral anticoagulation regimens without the risk of stroke.

I hope that you enjoy reading this issue of the *Journal.* Wishing you a relaxing summer.

Sincerely,


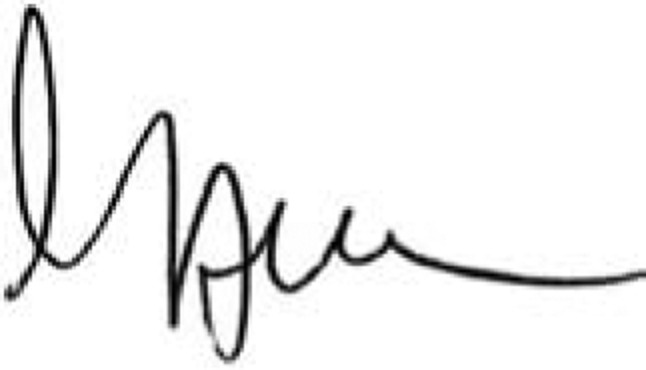


Moussa Mansour, MD, FHRS, FACC

Editor-in-Chief

The Journal of Innovations in Cardiac Rhythm Management

MMansour@InnovationsInCRM.com

Director, Atrial Fibrillation Program

Massachusetts General Hospital

Boston, MA 02114

